# Preoperative Serum Gamma-Glutamyltransferase as a Prognostic Biomarker in Patients Undergoing Radical Cystectomy for Bladder Cancer

**DOI:** 10.3389/fonc.2021.648904

**Published:** 2021-05-07

**Authors:** Shiqiang Su, Lizhe Liu, Chao Sun, Yanhua Nie, Hong Guo, Yang Hu, Shunli Guo, Shujian Pang

**Affiliations:** ^1^ Department of Urology, Shijiazhuang People’s Hospital, Shijiazhuang, China; ^2^ Institute of Medicine and Health, Hebei Medical University, Shijiazhuang, China

**Keywords:** bladder cancer, radical cystectomy, gamma-glutamyltransferase, prognosis, biomarkers

## Abstract

**Background:**

Serum gamma-glutamyltransferase (GGT) has been reported to be correlated with survival in a variety of malignancies. However, its effect on patients with bladder cancer (BC) treated by radical cystectomy has never been evaluated.

**Patients and Methods:**

We retrospectively evaluated 263 patients who underwent radical surgery in our center. Baseline features, hematologic variables, and follow-up data were obtained. The endpoints included overall survival (OS), cancer-specific survival (CSS), and disease-free survival (DFS). The relationship between GGT and survival were evaluated.

**Results:**

The median follow-up period for all patients was 34.7 (22.9-45.9) months. At the last follow-up, 67 patients died, 51 patients died of cancer, 92 patients experienced disease recurrence. Patients with an elevated serum GGT had a higher rate of pT3-T4 tumors. Patients with a higher preoperative serum GGT had a lower rate of OS, CSS and DFS (P < 0.001 for all). Multivariate analysis identified that preoperative serum GGT was independent predictor of OS (HR: 3.027, 95% CI: 1.716-5.338; P < 0.001), CSS (HR: 2.115, 95% CI: 1.093-4.090; P = 0.026), DFS (HR: 2.584, 95% CI: 1.569-4.255; P < 0.001). Age, diabetes history, pathologic T stage, and lymph node status also were independent predictors of prognosis for BC patients.

**Conclusions:**

Our results indicated that preoperative serum GGT was an independent prognosis predictor for survival of BC patients after radical cystectomy, and can be included in the prognostic models.

## Introduction

Bladder cancer (BC) is the second most common malignance of the urinary system. In 2020, there were estimated 81,400 new cases and 17,980 deaths of bladder cancer in the United States (1). Though most of newly diagnosed bladder cancers are presented with non-muscle-invasive disease, nearly 25% of them are muscle invasive BC. For non-muscle-invasive BC, though treating by transurethral tumor exsection, patients often experienced disease recurrence and progression. The rate of disease recurrence for non-muscle-invasive BC in 5 years ranges from 50% to 70%, and the 5-year progression rate ranges from 10% to 30% (2). For muscle invasive BC, after radical surgery and lymph node dissection, these patients have a poor long-term prognosis, with about a 50% for 5-year survival rate (3). Despite the significant progress in systematic therapy, the high rate of mortality for muscle invasive BC hasn’t changed. Since the common clinical staging system performed poor, more prognosis predictors are required to effectively classify BC patients for optimal treatment.

Due to the clinical convenience, several circulating biomarkers have been reported in bladder cancer prognosis, including lactate dehydrogenase (4), hemoglobin, white blood cell count, De Ritis ratio, albumin-globulin ratio, and so on (5). As a membrane bounding enzyme, serum gamma-glutamyltransferase (GGT) has been found to exert key value in glutathione metabolism and is crucial for cellular protection against various oxidants. The pathological state of oxidative stress, which can be observed in the various tumor microenvironment, has been found to result in an elevation in glutathione and GGT levels (6, 7). In addition, GGT is considered to play a potentially vital role in tumor development, disease progression, and anti-cancer drug resistance (7–10). Moreover, GGT has been found as an important prognostic biomarker in various cancer entities, including breast cancer (11), hepatocellular carcinoma (12), ovarian cancer (13), prostate cancer (14), renal cell carcinoma (15), and esophageal squamous cell carcinoma (16). However, to our knowledge, no study has looked at the role of GGT in bladder cancer prognosis. Hence, we conducted this study to assess the prognostic value of preoperative serum GGT in BC patients undergoing radical surgery.

## Materials and Methods

### Patient Selection

**Figure 1:**
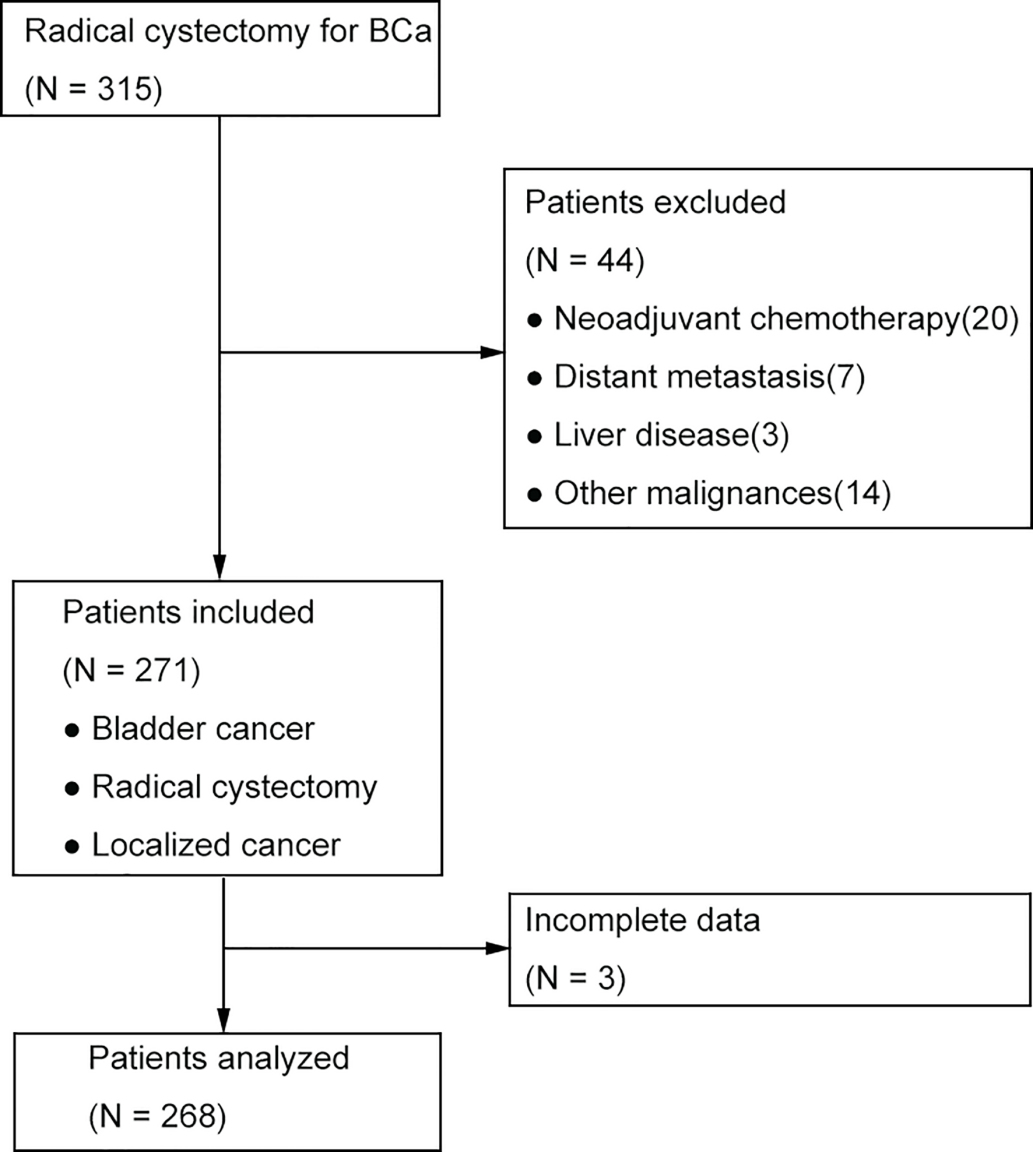
The flow chart for patient selection.

We retrospectively screened the medical records of bladder cancer in our hospital. Between January 2011 and December 2016, there were 315 cases with BC treated by open or laparoscopic radical cystectomy. Patients having the evidence of distant metastasis, undergoing neoadjuvant chemotherapy, having preoperative infection and liver diseases, accompanying other malignances were excluded. Lastly, after excluding 3 patients lacking key data, 268 patients were included for data analyzing (**Figure 1**). The study was approved by the institutional review board before it was carried out.

### Variables

These variables were extracted from medical records in our hospital. Preoperative variables consisted of demographics and hematological variables, including gender, age, body mass index, ASA score, history of smoking, history of common chronic diseases, serum hemoglobin, number of white blood cell, platelet to lymphocyte ratio (PLR), neutrophil to lymphocyte ratio (NLR), lymphocyte to monocyte ratio (LMR), serum albumin and GGT. The levels of hematological variables were examined by the most recent blood sample examinations conducted within 2 weeks prior to surgery. GGT was detected by IFCC method. After the reaction, the absorption peak was detected at 405nm to calculate the serum concentration of GGT. Postoperative variables consisted of surgical and pathological variables, including surgical approach and procedure, pT stage, status of lymph node, surgical margin, postoperative chemotherapy. The cut-off values of hemoglobin, white blood cell, PLR, NLR, LMR, albumin were decided according to our previous study (4). The best cut-off point of preoperative GGT was estimated through receiver operating characteristic (ROC) curve analysis, which was used as a classification variable to divide the whole cohort into two groups.

### Postoperative Management

Patient follow-up embraced regular clinical examinations and laboratory tests, imaging methods included chest radiography, abdominal/pelvic ultrasound, CT or MRI. Follow-up evaluations were conducted every three months for the first two years, semiannually for the next two years, and annually thereafter. Postoperative chemotherapy was administered for some patients when needed and accepted.

### Statistical Analysis

The primary outcome was overall survival (OS), the secondary outcomes included cancer-specific survival (CSS) and disease-free survival (DFS).

The normality of continuous variables (age and BMI) was checked by the Kolmogorov-Smirnov test. Due to the non-normal distribution, they were described as median and interquartile range. According to the cut-off value of GGT, all patients were divided into two groups. The associations between preoperative GGT and demographics, hematological, surgical, pathological variables were studied. For continuous variables, the Wilcoxon rank-sum test was applied. For classified variables, the Pearson *X* ^2^ or Fisher exact test was applied. The Kaplan-Meier method was applied to calculate patients’ clinical endpoints, and the differences between two groups were compared using the log-rank test. The univariate and multivariate Cox proportion analysis was performed to determine the effect of regular variables and preoperative GGT on OS, CSS and DFS. Hazard ratios (HRs) and 95% confidence intervals (CIs) were estimated by cox proportional analysis. Statistical analyses were conducted using SPSS 14.0 (Chicago, Illinois, USA). A two-sided P < 0.05 was deemed as statistically significant.

## Results

### Patient Characteristics

The baseline features of included patients were presented in **Table 1**. The median age at the time of operation was 63 (54-69) years. Of all patients, 41 (15.3%) were female, 227 (84.7%) were male. Seventy-eight (29.1%), forty-one (15.3%), and thirty-seven (13.8%) patients were respectively accompanied with hypertension, diabetes, and cardiac disease. Open and laparoscopic surgeries were performed in 104 (38.8%) and 164 (61.2%) patients, and 13 (4.9%) underwent neobladder, 206 (76.9%) underwent ileal conduit, 49 (18.3%) underwent continent cutaneous. Seventy-eight (29.1%) patients presented with pathological T3-T4 tumors, 40 (14.9%) patients presented with positive lymph node, 7 (2.6%) patients presented with positive surgical margin. Further postoperative chemotherapy was administered in 47 (17.5%) patients.

**Table 1:** Baseline characteristics of patients undergoing radical cystectomy for bladder cancer.

Variable	Overall	Normal GGT	Elevated GGT	P value
No. patients	268	220	48	
Age, years, median (IQR)	63 (54-69)	64 (54-70)	57.5 (53-67)	0.054
Gender, n (%)				0.662
Female	41 (15.3)	35 (15.9)	6 (12.5)	
Male	227 (84.7)	185 (84.1)	42 (87.5)	
BMI, kg/m^2^, median (IQR)	24.2 (22.5-26.4)	24.0 (22.5-26.1)	24.9 (22.4-27.6)	0.109
ASA score, n (%)				0.532
1	9 (3.4)	6 (2.7)	3 (6.3)	
2	220 (82.1)	182 (82.7)	38 (79.2)	
3	39 (14.6)	32 (14.5)	7 (14.6)	
Previous medical history, n (%)				
Hypertension	78 (29.1)	61 (27.7)	17 (35.4)	0.297
Diabetes	41 (15.3)	32 (14.5)	9 (18.8)	0.507
Cardiac disease	37 (13.8)	30 (13.6)	7 (14.6)	1.000
Smoker, n (%)				0.143
None	139 (51.9)	119 (54.1)	20 (41.7)	
Present	106 (39.6)	81 (36.8)	25 (52.1)	
Former	23 (8.6)	20 (9.1)	3 (6.3)	
Surgical approach, n (%)				0.872
Open	104 (38.8)	86 (39.1)	18 (37.5)	
Laparoscopic	164 (61.2)	134 (60.9)	30 (62.5)	
Urinary diversion type, n (%)				0.535
Neobladder	13 (4.9)	11 (5.0)	2 (4.2)	
Ileal conduit	206 (76.9)	166 (75.5)	40 (83.3)	
Continent cutaneous	49 (18.3)	43 (19.5)	6 (12.5)	
Pathologic T stage, n (%)				0.037
T1-T2	190 (70.9)	162 (73.6)	28 (58.3)	
T3-T4	78 (29.1)	58 (26.4)	20 (41.7)	
Lymph node status, n (%)				0.716
pN0	200 (74.6)	166 (75.5)	34 (70.8)	
pN+	40 (14.9)	31 (14.1)	9 (18.8)	
pNx	28 (10.4)	23 (10.5)	5 (10.4)	
Surgical margin status, n (%)				0.612
Negative	261 (97.4)	215 (97.7)	46 (95.8)	
Positive	7 (2.6)	5 (2.3)	2 (4.2)	
Postoperative chemotherapy, n (%)				0.531
Absence	221 (82.5)	183 (83.2)	38 (79.2)	
Presence	47 (17.5)	37 (16.8)	10 (20.8)	
Preoperative anemia, n (%)				0.075
No	194 (72.4)	154 (70.0)	40 (83.3)	
Yes	74 (27.6)	66 (30.0)	8 (16.7)	
White blood cell, n (%)				1.000
Normal	251 (93.7)	206 (93.6)	45 (93.8)	
Elevated	17 (6.3)	14 (6.4)	3 (6.3)	
NLR, n (%)				0.542
≤3	217 (81.0)	180 (81.8)	37 (77.1)	
>3	51 (19.0)	40 (18.2)	11 (22.9)	
PLR, n (%)				1.000
≤150	252 (94.0)	207 (94.1)	45 (93.8)	
>150	16 (6.0)	13 (5.9)	3 (6.3)	
LMR, n (%)				0.375
≤3.5	70 (26.1)	60 (27.3)	10 (20.8)	
>3.5	198 (73.9)	160 (72.7)	38 (79.2)	
Albumin, g/dl, n (%)				0.699
≥4	256 (95.5)	209 (95.0)	47 (97.9)	
<4	12 (4.5)	11 (5.0)	1 (2.1)	

GGT, gamma-glutamyltransferase; IQR, interquartile range; BMI, body mass index; ASA, American Society of Anesthesiologists; NLR, neutrophil-to-lymphocyte ratio; PLR, platelet-to-lymphocyte ratio; LMR, lymphocyte-to-monocyte ratio.

The median follow-up period for all subjects was 34.7 (22.9-45.9) months. At the last follow-up, 67 patients died, 51 patients died of cancer, 92 patients experienced disease recurrence. The three-year OS, CSS, DFS was respectively 74.5%, 78.8%, 66.8%. The five-year OS, CSS, DFS was respectively 61.6%, 67.9%, 51.3%.

### Preoperative Serum GGT and Clinicopathological Parameters

The optimal cut-off point of preoperative GGT was 40 U/L. Forty-eight patients had an elevated level of serum GGT, a normal level of serum GGT was presented in 220 patients. Between the two groups, we only found that patients with an elevated serum GGT had a higher rate of pT3-T4 tumors (41.7% vs. 26.4%, P = 0.037). However, the other variables including demographics, hematological, surgical, pathological variables were similar between the two groups (P > 0.05 for all).

### Preoperative Serum GGT With Survival

**Figure 2:**
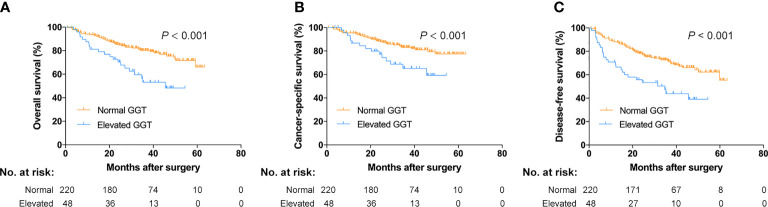
Survival curves for normal and elevated GGT groups. **(A)** Overall survival; **(B)** cancer-specific survival; **(C)** disease-free survival.

The Kaplan-Meier curves were presented in **Figure 2**. According to the results, patients with a higher preoperative serum GGT had a lower rate of OS, CSS and DFS (P < 0.001 for all).

Older age (P = 0.001), diabetes history (P = 0.017), continent cutaneous urinary diversion (P = 0.044), higher pT stage (P < 0.001), positive lymph node (P < 0.001), postoperative chemotherapy (P = 0.014), higher level of NLR (P = 0.007), elevated level of preoperative serum GGT (P = 0.001) were correlated to lower rate of OS. Multivariate analysis identified that age (HR: 1.053, 95% CI: 1.022-1.085; P = 0.001), diabetes history (HR: 1.884, 95% CI: 1.024-3.466; P = 0.042), pathologic T stage (HR: 3.140, 95% CI: 1.772-5.564; P < 0.001), positive lymph node (HR: 1.946, 95% CI: 1.020-3.715; P = 0.043), preoperative serum GGT (HR: 3.027, 95% CI: 1.716-5.338; P < 0.001) were independent predictors of OS (**Table 2**).

**Table 2:** Univariate and multivariate analysis for variables predicting overall survival.

Variable	Univariate		Multivariate	
	HR (95% CI)	P value	HR (95% CI)	P value
Age, years	1.047 (1.020-1.074)	0.001	1.053 (1.022-1.085)	0.001
Gender		0.804		
Male (vs. female)	0.921 (0.482-1.761)			
BMI, kg/m^2^	0.969 (0.914-1.027)	0.288		
ASA score		0.191		
1	Reference			
2	1.140 (0.277-4.690)	0.856		
3	1.958 (0.445-8.623)	0.374		
Previous medical history				
Hypertension	0.980 (0.576-1.668)	0.942		
Diabetes	2.020 (1.134-3.598)	0.017	1.884 (1.024-3.466)	0.042
Cardiac disease	1.121 (0.572-2.196)	0.739		
Smoker		0.571		
None	Reference			
Present	0.914 (0.553-1.510)	0.725		
Former	0.575 (0.205-1.615)	0.294		
Surgical approach		1.000		
Laparoscopic (vs. open)	1.000 (0.612-1.634)			
Urinary diversion type		<0.001		0.045
Neobladder	Reference		Reference	
Ileal conduit	1.415 (0.342-5.848)	0.632	0.747 (0.175-3.186)	0.693
Continent cutaneous	4.415 (1.040-18.749)	0.044	1.586 (0.347-7.247)	0.552
Pathologic T stage		<0.001		<0.001
T3-T4 (vs. T1-T2)	4.348 (2.672-7.077)		3.140 (1.772-5.564)	
Lymph node status		<0.001		0.122
pN0	Reference		Reference	
pN+	3.588 (2.103-6.121)	<0.001	1.946 (1.020-3.715)	0.043
pNx	1.593 (0.742-3.418)	0.232	1.034 (0.457-2.340)	0.936
Surgical margin status		0.377		
Positive (vs. negative)	1.686 (0.529-5.377)			
Postoperative chemotherapy		0.014		0.691
Presence (vs. absence)	1.971 (1.147-3.386)		1.142 (0.594-2.195)	
Preoperative anemia		0.131		
Yes (vs. no)	1.471 (0.892-2.425)			
White blood cell		0.460		
Elevated (vs. normal)	0.646 (0.203-2.059)			
NLR		0.007		0.441
>3 (vs. ≤3)	2.046 (1.211-3.458)		1.251 (0.707-2.215)	
PLR		0.942		
>150 (vs. ≤150)	1.039 (0.378-2.857)			
LMR		0.314		
>3.5 (vs. ≤3.5)	0.767 (0.457-1.286)			
Albumin, g/dl		0.592		
<4 (vs. ≥4)	0.680 (0.166-2.780)			
GGT, U/l		0.001		<0.001
>40 (vs. ≤40)	2.454 (1.458-4.132)		3.027 (1.716-5.338)	

HR, hazard ratio; CI, confidence interval; BMI, body mass index; ASA, American Society of Anesthesiologists; NLR, neutrophil-to-lymphocyte ratio; PLR, platelet-to-lymphocyte ratio; LMR, lymphocyte-to-monocyte ratio; GGT, gamma-glutamyltransferase.

The results showed that diabetes history (P = 0.025), higher pT stage (P < 0.001), positive lymph node (P < 0.001), postoperative chemotherapy (P = 0.002), higher level of NLR (P = 0.011), elevated level of preoperative serum GGT (P = 0.012) were correlated to lower rate of CSS. Multivariate analysis identified that diabetes history (HR: 2.250, 95% CI: 1.126-4.496; P = 0.022), pathologic T stage (HR: 3.030, 95% CI: 1.574-5.834; P = 0.001), positive lymph node (HR: 2.555, 95% CI: 1.243-5.254; P = 0.011), preoperative serum GGT (HR: 2.115, 95% CI: 1.093-4.090; P = 0.026) were independent predictors of CSS (**Table 3**).

**Table 3:** Univariate and multivariate analysis for variables predicting cancer-specific survival.

Variable	Univariate		Multivariate	
	HR (95% CI)	P value	HR (95% CI)	P value
Age, years	1.026 (0.997-1.056)	0.077		
Gender		0.895		
Male (vs. female)	0.951 (0.447-2.022)			
BMI, kg/m^2^	0.968 (0.906-1.034)	0.333		
ASA score		0.107		
1	Reference			
2	1.746 (0.240-12.731)	0.582		
3	3.379 (0.439-26.004)	0.242		
Previous medical history				
Hypertension	1.026 (0.561-1.873)	0.935		
Diabetes	2.098 (1.097-4.011)	0.025	2.250 (1.126-4.496)	0.022
Cardiac disease	1.355 (0.659-2.784)	0.408		
Smoker		0.413		
None	Reference			
Present	1.056 (0.601-1.855)	0.850		
Former	0.396 (0.094-1.666)	0.207		
Surgical approach		0.595		
Laparoscopic (vs. open)	1.168 (0.660-2.065)			
Urinary diversion type		<0.001		0.003
Neobladder	Reference		Reference	
Ileal conduit	2.112 (0.288-15.466)	0.462	1.438 (0.193-10.717)	0.723
Continent cutaneous	6.811 (0.909-51.052)	0.062	4.303 (0.557-33.269)	0.162
Pathologic T stage		<0.001		0.001
T3-T4 (vs. T1-T2)	5.085 (2.889-8.950)		3.030 (1.574-5.834)	
Lymph node status		<0.001		0.020
pN0	Reference		Reference	
pN+	4.249 (2.380-7.568)	<0.001	2.555 (1.243-5.254)	0.011
pNx	0.797 (0.243-2.617)	0.708	0.640 (0.190-2.156)	0.471
Surgical margin status		0.159		
Positive (vs. negative)	2.313 (0.720-7.435)			
Postoperative chemotherapy		0.002		0.941
Presence (vs. absence)	2.496 (1.381-4.512)		0.973 (0.468-2.024)	
Preoperative anemia		0.133		
Yes (vs. no)	1.545 (0.875-2.728)			
White blood cell		0.767		
Elevated (vs. normal)	0.838 (0.261-2.693)			
NLR		0.011		0.554
>3 (vs. ≤3)	2.157 (1.193-3.901)		1.211 (0.642-2.284)	
PLR		0.570		
>150 (vs. ≤150)	1.345 (0.484-3.737)			
LMR		0.382		
>3.5 (vs. ≤3.5)	0.768 (0.425-1.388)			
Albumin, g/dl		0.873		
<4 (vs. ≥4)	0.891 (0.217-3.666)			
GGT, U/l		0.012		0.026
>40 (vs. ≤40)	2.172 (1.187-3.975)		2.115 (1.093-4.090)	

HR, hazard ratio; CI, confidence interval; BMI, body mass index; ASA, American Society of Anesthesiologists; NLR, neutrophil-to-lymphocyte ratio; PLR, platelet-to-lymphocyte ratio; LMR, lymphocyte-to-monocyte ratio; GGT, gamma-glutamyltransferase.

The results showed that older age (P = 0.007), higher pT stage (P < 0.001), positive lymph node (P < 0.001), positive surgical margin (P = 0.043), postoperative chemotherapy (P < 0.001), elevated level of preoperative serum GGT (P < 0.001) were associated with lower rate of DFS. Multivariate analysis identified that age (HR: 1.041, 95% CI: 1.016-1.066; P = 0.001), pathologic T stage (HR: 2.591, 95% CI: 1.580-4.250; P < 0.001), positive lymph node (HR: 1.954, 95% CI: 1.104-3.460; P = 0.022), preoperative serum GGT (HR: 2.584, 95% CI: 1.569-4.255; P < 0.001) were independent predictors of DFS (**Table 4**).

**Table 4:** Univariate and multivariate analysis for variables predicting progression-free survival.

Variable	Univariate		Multivariate	
	HR (95% CI)	P value	HR (95% CI)	P value
Age, years	1.030 (1.008-1.052)	0.007	1.041 (1.016-1.066)	0.001
Gender		0.691		
Male (vs. female)	0.894 (0.513-1.555)			
BMI, kg/m^2^	0.979 (0.930-1.030)	0.404		
ASA score		0.109		
1	Reference			
2	1.673 (0.410-6.826)	0.473		
3	2.748 (0.640-11.805)	0.174		
Previous medical history				
Hypertension	0.881 (0.557-1.395)	0.590		
Diabetes	1.505 (0.888-2.552)	0.129		
Cardiac disease	1.023 (0.569-1.840)	0.939		
Smoker		0.851		
None	Reference			
Present	0.895 (0.578-1.386)	0.618		
Former	1.052 (0.516-2.141)	0.890		
Surgical approach		0.196		
Laparoscopic (vs. open)	1.331 (0.863-2.054)			
Urinary diversion type		<0.001		0.098
Neobladder	Reference		Reference	
Ileal conduit	1.277 (0.401-4.074)	0.679	0.753 (0.231-2.452)	0.638
Continent cutaneous	3.207 (0.974-10.553)	0.055	1.313 (0.372-4.633)	0.672
Pathologic T stage		<0.001		<0.001
T3-T4 (vs. T1-T2)	4.014 (2.648-6.085)		2.591 (1.580-4.250)	
Lymph node status		<0.001		0.071
pN0	Reference		Reference	
pN+	4.226 (2.656-6.725)	<0.001	1.954 (1.104-3.460)	0.022
pNx	1.905 (1.036-3.504)	0.038	1.169 (0.616-2.218)	0.633
Surgical margin status		0.043		0.645
Positive (vs. negative)	2.545 (1.028-6.301)		1.245 (0.490-3.160)	
Postoperative chemotherapy		<0.001		0.061
Presence (vs. absence)	2.898 (1.870-4.492)		1.683 (0.976-2.902)	
Preoperative anemia		0.305		
Yes (vs. no)	1.257 (0.812-1.945)			
White blood cell		0.187		
Elevated (vs. normal)	0.461 (0.146-1.458)			
NLR		0.088		
>3 (vs. ≤3)	1.519 (0.940-2.456)			
PLR		0.540		
>150 (vs. ≤150)	0.731 (0.268-1.991)			
LMR		0.388		
>3.5 (vs. ≤3.5)	0.821 (0.524-1.286)			
Albumin, g/dl		0.635		
<4 (vs. ≥4)	0.757 (0.239-2.392)			
GGT, U/l		<0.001		<0.001
>40 (vs. ≤40)	2.329 (1.476-3.676)		2.584 (1.569-4.255)	

HR, hazard ratio; CI, confidence interval; BMI, body mass index; ASA, American Society of Anesthesiologists; NLR, neutrophil-to-lymphocyte ratio; PLR, platelet-to-lymphocyte ratio; LMR, lymphocyte-to-monocyte ratio; GGT, gamma-glutamyltransferase.

## Discussion

Despite the significant progress in systematic therapy, the long-term survival hasn’t changed significantly for BC patients, especially cases with muscle-invasive disease. At present, biomarkers with prognostic significance in bladder cancer include cell cycle regulating proteins, cell adhesion molecules, oncogenes, tumor related antigens, etc (17). Nevertheless, in order to better understand the mechanism of disease occurrence and progression, more dependable prognosis predictors are still needed. A lot of researches have explored the prognostic value of serum GGT in various cancer patients. However, to our knowledge, no study has looked at the role of GGT in bladder cancer prognosis. Hence, we conducted this study to assess the prognostic value of preoperative serum GGT in BC patients undergoing radical surgery.

In patients with urological cancers, Takemura et al. (14) have assessed the prognostic value of serum GGT on mCRPC patients treated with enzalutamide and its correlation with treatment response. They found that an increase in serum GGT was significantly and independently associated with a shorter overall survival. An elevation in GGT was also correlated to a poorer PSA response, the largest PSA change, and a shorter PSA progression-free survival. Luo et al. (15) have assessed the prognostic value of preoperative serum GGT in the subgroup renal cell patients with tumor thrombus. They found that preoperative elevation of GGT was correlated to poor CSS and recurrence-free survival, and the preoperative GGT level can be an important prognosis predictor for patient with localized renal cell carcinoma and tumor thrombus.

To our best knowledge, we initially examined the prognostic significance of preoperative serum GGT in BC patients. We found that patients with an increased serum GGT had a higher rate of pT3-T4 tumors (41.7% vs. 26.4%, P = 0.037). Since high pathological T stage tumors experienced unfavorable oncological outcomes (4), an increase in GGT level may associated with an inferior prognosis in BC patients. All subjects were classified into two groups based on the cut-off point of preoperative GGT at 40 U/L, the Kaplan-Meier curves were performed and verified that patients belonging to high serum GGT group had a lower rate of OS, CSS and DFS (P < 0.001 for all). Moreover, including other demographics, hematological, surgical and pathological variables, cox regression analyses have found that preoperative serum GGT was an independent predictor for OS (HR: 3.027, 95% CI: 1.716-5.338; P < 0.001), CSS (HR: 2.115, 95% CI: 1.093-4.090; P = 0.026), and DFS (HR: 2.584, 95% CI: 1.569-4.255; P < 0.001) in BC patients undergoing radical cystectomy. Circulating serum GGT is clinically easy to detect and can be applied as a viewer of cancer burden and a meaningful marker in clinical treatment of BC patients.

As a membrane-bound enzyme, GGT plays an important role in maintaining the production of intracellular GSH, an important antioxidant that protects cells from reactive oxygen compounds and free radicals. GGT also plays an important role in supplying amino acids to cells and promoting cell proliferation both physiologically and by limiting cysteine concentrations (7). There is growing evidence that GGT is deregulated in tumor cells and can represent cancer progression and aggressiveness (18). It was proposed that the increase of GGT may contribute to the formation of tumor microenvironment and protect cancer cells from oxidative stress or cytotoxic drugs (19). Nevertheless, GGT can also promote oxidative effects in certain situations. Persistent oxidative stress leads to genomic instability and following imbalance of cell proliferation and apoptosis, which is involved in carcinogenesis and progression (9). Hence, GGT can be used as an indicator of tumor aggressiveness by presenting the degree of oxidative stress. Additionally, it has been reported that GGT can be induced by a variety of inflammatory factors, including tumor necrosis factor and interferon (20, 21). Therefore, we hypothesized that GGT was associated with tumor-associated inflammatory responses and may serve as an inflammatory biomarker to predict the prognosis of cancer patients. Nevertheless, the exact mechanism of increased GGT level in the process of tumor formation was not clearly clarified.

In our study, we also found that age, diabetes history, pathologic T stage, and positive lymph status were independent predictors of prognosis for BC patients undergoing radical cystectomy. Although the difference was significant for the variable age, the predictive effect (HR: 1.053, 95% CI: 1.022-1.085) was minor. The recent publication has reported the similar result (HR: 1.03, 95% CI: 1.01-1.05) (22). In 2018, Peng et al. (23) have performed an updated meta-analysis to examine the relationship between metabolic syndrome and susceptibility and prognosis of bladder cancer. The pooled results indicated that diabetes history significantly increased the risk of bladder cancer and was associated with poor survival. More recently, a Chinese cohort has explored the prognostic value of CD155 in patients with localized muscle-invasive BC. In multivariate analysis, they also identified that T stage and positive lymph node independently predicted relapse-free survival and overall survival. They have further verified that T stage and lymph node status were independent predictors associated with inferior overall survival with data from TCGA database (24). The serum GGT reminded to be an independent predictor in our study after adjusting for these common prognostic factors.

The limitations can’t be ignored. First, this was a single-institutional retrospective study with limited patients, the potential selection bias can’t be controlled. Second, because of the retrospective design, some data may miss and be collected inaccurately, which may affect the results. Third, the median follow-up period for all patients was 34.7 months, which may be insufficient, especially for patients at low risk. Fourth, the cut-off value of serum GGT has not been determined. Through ROC curve analysis, the cut-off point of preoperative GGT was 40 U/L in our study. The best cut-off value of serum GGT reminds to be explored. Thus, prospective external validation from well-designed independent multi-institution cohorts with sufficient follow-up time will verify the universality of our results.

## Conclusions

Despite the above limitations, the present study initially investigated the prognostic value of preoperative GGT in BC patients. Our results indicated that preoperative serum GGT was an independent prognosis predictor for survival of BC patients after radical surgery, and can be included in the prognostic models.

## Data Availability Statement

The original contributions presented in the study are included in the article/supplementary material, further inquiries can be directed to the corresponding authors.

## Ethics Statement

The studies involving human participants were reviewed and approved by The Ethics Committe of Shijiazhuang People’s Hospital. The patients/participants provided their written informed consent to participate in this study.

## Author Contributions

Conception and design: SS and CS. Data collection or management: SS, LL, YN, and HG. Data analysis: SS, YH, SG, and SP. Manuscript writing/editing: SS and CS. All authors contributed to the article and approved the submitted version.

## Funding

This work was supported by a grant from science and technology research and development guidance project of Shijiazhuang in 2020 (No. 201460663).

## Conflict of Interest

The authors declare that the research was conducted in the absence of any commercial or financial relationships that could be construed as a potential conflict of interest.

## References

[1] Siegel RL Miller KD Jemal A. Cancer Statistics, 2020. CA: Cancer J Clin (2020) 70(1):7–30. 10.3322/caac.21590 31912902

[2] Kamat AM Hahn NM Efstathiou JA Lerner SP Malmström PU Choi W. Bladder Cancer. Lancet (2016) 388(10061):2796–810. 10.1016/S0140-6736(16)30512-8 27345655

[3] Su S Gu L Ma X Li H Wang B Shi T. Comparison of Laparoscopic and Robot-assisted Radical Cystectomy for Bladder Cancer: Perioperative and Oncologic Outcomes. Clin Genitourin Cancer (2019) 17(5):e1048-e53. 10.1016/j.clgc.2019.06.007 31303560

[4] Su S Liu L Sun C Yang L Nie Y Chen Y. Prognostic Significance of Serum Lactate Dehydrogenase in Patients Undergoing Radical Cystectomy for Bladder Cancer. Urol Oncol (2020) 38(11):852.e1–9. 10.1016/j.urolonc.2020.05.031 32624424

[5] Mori K Miura N Mostafaei H Quhal F Motlagh RS Lysenko I. Prognostic Value of Preoperative Hematologic Biomarkers in Urothelial Carcinoma of the Bladder Treated With Radical Cystectomy: A Systematic Review and Meta-Analysis. Int J Clin Oncol (2020) 25(8):1459–74. 10.1007/s10147-020-01690-1 PMC7392936 32451768

[6] Whitfield JB. Gamma Glutamyl Transferase. Crit Rev Clin Lab Sci (2001) 38(4):263–355. 10.1080/20014091084227 11563810

[7] Hanigan MH Gallagher BC Townsend DM Gabarra V. Gamma-Glutamyl Transpeptidase Accelerates Tumor Growth and Increases the Resistance of Tumors to Cisplatin In Vivo. Carcinogenesis (1999) 20(4):553–9. 10.1093/carcin/20.4.553 PMC6522259 10223181

[8] Van Hemelrijck M Jassem W Walldius G Fentiman IS Hammar N Lambe M. Gamma-Glutamyltransferase and Risk of Cancer in a Cohort of 545,460 Persons - the Swedish AMORIS Study. Eur J Cancer (2011) 47(13):2033–41. 10.1016/j.ejca.2011.03.010 21486691

[9] Corti A Franzini M Paolicchi A Pompella A. Gamma-Glutamyltransferase of Cancer Cells At the Crossroads of Tumor Progression, Drug Resistance and Drug Targeting. Anticancer Res (2010) 30(4):1169–81. 20530424

[10] Pompella A Corti A Paolicchi A Giommarelli C Zunino F. Gamma-Glutamyltransferase, Redox Regulation and Cancer Drug Resistance. Curr Opin Pharmacol (2007) 7(4):360–6. 10.1016/j.coph.2007.04.004 17613273

[11] Staudigl C Concin N Grimm C Pfeiler G Nehoda R Singer CF. Prognostic Relevance of Pretherapeutic Gamma-Glutamyltransferase in Patients With Primary Metastatic Breast Cancer. PloS One (2015) 10(4):e0125317. 10.1371/journal.pone.0125317 25915044 PMC4411095

[12] Xu XS Wan Y Song SD Chen W Miao RC Zhou YY. Model Based on γ-Glutamyltransferase and Alkaline Phosphatase for Hepatocellular Carcinoma Prognosis. World J Gastroenterol (2014) 20(31):10944–52. 10.3748/wjg.v20.i31.10944 PMC4138475 25152598

[13] Grimm C Hofstetter G Aust S Mutz-Dehbalaie I Bruch M Heinze G. Association of Gamma-Glutamyltransferase With Severity of Disease At Diagnosis and Prognosis of Ovarian Cancer. Br J Cancer (2013) 109(3):610–4. 10.1038/bjc.2013.323 PMC3738124 23921280

[14] Takemura K Ito M Nakanishi Y Kataoka M Sakamoto K Suzuki H. Serum γ-Glutamyltransferase as a Prognostic Biomarker in Metastatic Castration-Resistant Prostate Cancer Treated With Enzalutamide. Anticancer Res (2019) 39(10):5773–80. 10.21873/anticanres.13780 31570481

[15] Luo C Xu B Fan Y Yu W Zhang Q Jin J. Preoperative Gamma-Glutamyltransferase is Associated With Cancer-Specific Survival and Recurrence-Free Survival of Nonmetastatic Renal Cell Carcinoma With Venous Tumor Thrombus. BioMed Res Int (2017) 2017:3142926. 10.1155/2017/3142926 28168196 PMC5266806

[16] Yang F Zhang S Yang H Luo K Wen J Hu Y. Prognostic Significance of Gamma-Glutamyltransferase in Patients With Resectable Esophageal Squamous Cell Carcinoma. Dis Esophagus (2015) 28(5):496–504. 10.1111/dote.12227 24766310

[17] Ku JH Kim WJ Lerner SP Chun F Kluth LA. Diagnostic and Prognostic Markers in Bladder Cancer. Dis Markers (2016) 2016:2425091. 10.1155/2016/2425091 27795607 PMC5067309

[18] Strasak AM Goebel G Concin H Pfeiffer RM Brant LJ Nagel G. Prospective Study of the Association of Serum Gamma-Glutamyltransferase With Cervical Intraepithelial Neoplasia III and Invasive Cervical Cancer. Cancer Res (2010) 70(9):3586–93. 10.1158/0008-5472.CAN-09-3197 PMC5911687 20388786

[19] Franzini M Corti A Lorenzini E Paolicchi A Pompella A De Cesare M. Modulation of Cell Growth and Cisplatin Sensitivity by Membrane Gamma-Glutamyltransferase in Melanoma Cells. Eur J Cancer (2006) 42(15):2623–30. 10.1016/j.ejca.2006.04.016 16928443

[20] Bouman L Sancéau J Rouillard D Bauvois B. gamma-Glutamyl Transpeptidase Expression in Ewing’s Sarcoma Cells: Up-Regulation by Interferons. Biochem J (2002) 364(Pt 3):719–24. 10.1042/bj20011854 PMC1222621 12049636

[21] Daubeuf S Accaoui MJ Pettersen I Huseby NE Visvikis A Galteau MM. Differential Regulation of Gamma-Glutamyltransferase mRNAs in Four Human Tumour Cell Lines. Biochim Biophys Acta (2001) 1568(1):67–73. 10.1016/S0304-4165(01)00201-X 11731087

[22] Lai C Wu Z Shi J Li K Zhu J Chen Z. Autophagy-Related Long Noncoding RNAs can Predict Prognosis in Patients With Bladder Cancer. Aging (Albany NY) (2020) 12(21):21582–96. 10.18632/aging.103947 PMC7695412 33175697

[23] Peng XF Meng XY Wei C Xing ZH Huang JB Fang ZF. The Association Between Metabolic Syndrome and Bladder Cancer Susceptibility and Prognosis: An Updated Comprehensive Evidence Synthesis of 95 Observational Studies Involving 97,795,299 Subjects. Cancer Manag Res (2018) 10:6263–74. 10.2147/CMAR.S181178 PMC6267767 30568489

[24] Zhang J Zhu Y Wang Q Kong Y Sheng H Guo J. Poliovirus Receptor CD155 is Up-Regulated in Muscle-Invasive Bladder Cancer and Predicts Poor Prognosis. Urol Oncol (2020) 38(2):41.e11–8. 10.1016/j.urolonc.2019.07.006 31383549

